# Phenolic Content, Antioxidant Activity and In Vitro Anti-Inflammatory and Antitumor Potential of Selected Bulgarian Propolis Samples

**DOI:** 10.3390/biomedicines13020334

**Published:** 2025-02-01

**Authors:** Yulian Tumbarski, Ivan Ivanov, Mina Todorova, Sonia Apostolova, Rumiana Tzoneva, Krastena Nikolova

**Affiliations:** 1 Department of Microbiology and Biotechnology, University of Food Technologies, 4002 Plovdiv, Bulgaria; 2 Department of Organic Chemistry and Inorganic Chemistry, University of Food Technologies, 4002 Plovdiv, Bulgaria; ivanov_ivan.1979@yahoo.com; 3 Department of Organic Chemistry, Paisii Hilendarski University of Plovdiv, 4000 Plovdiv, Bulgaria; mm_todorova@abv.bg; 4 Laboratory of Transmembrane Signaling, Institute of Biophysics and Biomedical Engineering, Bulgarian Academy of Sciences, 1113 Sofia, Bulgaria; sonia_apostolova@yahoo.com (S.A.); rtzoneva65@gmail.com (R.T.); 5 Department of Physics and Biophysics, Medical University—Varna, 9000 Varna, Bulgaria

**Keywords:** propolis, phenolic profile, antioxidant activity, anti-inflammatory activity, antitumor activity

## Abstract

Background/objectives: Propolis (bee glue) is a valuable bee product widely used as a natural remedy, a cosmetic ingredient, a nutritional value enhancer and a food biopreservative. The present research aims to investigate the phenolic content, antioxidant activity and in vitro anti-inflammatory and antitumor potential of six propolis samples from three regions of Bulgaria (Vidin, Gabrovo and Lovech). Methods: the analysis of propolis phenolic compounds was determined by high-performance liquid chromatography (HPLC); the antioxidant activity of ethanolic propolis extracts was assessed by the 2,2-diphenyl-1-picrylhydrazyl (DPPH) radical scavenging assay and ferric-reducing antioxidant power (FRAP) assay; the in vitro anti-inflammatory activity was assessed by the inhibition of albumin denaturation method; the in vitro antitumor activity was determined in human metastatic breast cancer cell line MDA-MB-231 using 3-(4,5-Dimethyl -2-thiazolyl)-2,5-diphenyl-2H-tetrazolium bromide (MTT) assay. Results: The ethanolic propolis extracts exhibited the total phenolic content from 190.4 to 317.0 mg GAE/g, total flavonoid content from 53.4 to 79.3 mg QE/g and total caffeic acid derivatives content from 5.9 to 12.1 mg CAE/g. The studied propolis extracts showed significant antioxidant capacity (between 1000.3 and 1606.0 mM TE/g determined by the DPPH assay, and between 634.1 and 1134.5 mM TE/g determined by the FRAP assay). The chemical composition analysis indicated high concentrations of caffeic acid benzyl ester, chrysin, pinocembrin and pinobanksin-3-O-propionate, predominantly in three of the propolis samples originating from Gabrovo and Lovech regions. All propolis samples demonstrated promising in vitro anti-inflammatory activity, expressed as the inhibition of thermally induced albumin denaturation by 73.59% to 78.44%, which was higher than that of the conventional anti-inflammatory drugs Aspirin (58.44%) and Prednisolone Cortico (57.34%). The propolis samples exhibited high in vitro cytotoxicity against cancer cells MDA-MB-231 with IC_50_ values ranging between 9.24 and 13.62 µg/mL as determined by MTT assay. Conclusions: Overall, we can suggest that the high phenolic content of Bulgarian propolis from respective areas contributes to its enhanced antioxidant, anti-inflammatory and antitumor activity. Taken together, our results support the beneficial properties of Bulgarian propolis, with potential application as a promising therapeutic agent.

## 1. Introduction

Propolis (bee glue) is a beekeeping product, which has been known by people since antiquity. Its extensive application as a remedy and a cosmetic ingredient dates back to the times of the ancient Greeks, Romans, Persians and Egyptians, who used its medicinal properties for the first time. Propolis is a complex biological substance produced by European honeybees (*Apis mellifera* L.) by collecting exudates from the flowers and leaf buds of various plant species. Worker bees collect the resins with their mandibles, transport them to the hive on their hind legs and process them with saliva and wax. Due to its highly adhesive properties, bees use propolis to fill the cracks and smooth out the internal walls, thus controlling the air flow, temperature and moisture in the hive. Another important function of propolis as a plastic material is to repair and seal up the cells of the honeycomb. Besides its role as a building material, it serves as an effective antimicrobial agent through whom bees protect their colony from infections by embalming the carcasses of invaders, who had penetrated and died inside their hives, thus eliminating the harmful microflora and the unpleasant odor accompanying their decomposition [1].

Propolis represents a complex biological substance possessing certain chemical composition and biological properties that are closely related to the type of plant source and the characteristics of the geographic region from which it originates. According to some recent studies, the number of chemical compounds identified as constituents of propolis continues to grow. The phenolic compounds are reported as the main components of propolis, which are responsible for most of its biological activities. Fatty acids, fatty acid esters, amino acids, enzymes, sugars, vitamins, minerals and pollen are also recognized as propolis ingredients [2]. The main source of poplar-type (Eurasian) propolis are the bud exudates of *Populus* spp. and their hybrids, containing pinocembrin, pinobanksin 3-O-acetate, galangin, chrysin, pentenyl caffeates, phenethyl caffeate, caffeic acid, ferulic acid and isoferulic acid as major bioactive components. The main source of Brazilian green propolis are the exudates secreted by *Baccharis dracunculifolia*; therefore, it contains baccharin, druparin, aromadendrine, artepillin C and caffeoylquinic acids, while the source of red propolis originating from Brazil, Cuba and Mexico are the exudates of *Dalbergia* spp., which comprise vestitol, 3-O-methyl vestitol and medicarpin as principal constituents. The resinoids of coniferous plants of the *Cupressaceae* family are the main source of propolis originating from the Mediterranean region, which contains diterpenes as its main phytochemical compounds and limited phenolic content [3,4].

Depending on the territorial manifestation of the climatic factors, Bulgaria is divided into five climatic regions (temperate continental, transitional continental, continental Mediterranean, Black Sea and mountainous), each of which is characterized by exceptional soil and botanical diversity. Previous studies revealed that Bulgarian propolis includes phenolic compounds (mainly caffeic acid and respective derivatives—pentenyl caffeates, benzyl phenethyl caffeates) and flavonoids (pinocembrin, pinobanksin, pinobanksin O-aceteate, chrysin, galangin) as its major constituents, which are typical for the poplar-type propolis [5,6].

The rich chemical composition of propolis determines its wide range of pharmacological effects. According to numerous studies, it has been found that propolis exhibits antioxidant, antibacterial, antifungal, anti-inflammatory, antitumor, wound-healing [7], antiviral [8], antiparasitic [9], immuno-modulatory [10], anesthetic [11], antidiabetic, antihyperlipidemic, anti-obesity [12], gastroprotective and gastric-healing [13] properties, among the others, making this beekeeping product a valuable remedy in apitherapy and other branches of medicine. Propolis is a natural and safe biological substance with significant antimicrobial and antioxidant potential, which has been utilized in the food and nutraceutical industries as a promising food biopreservative, a food packaging material and a functional food ingredient [14,15].

The present study aimed to investigate the phenolic profile, antioxidant activity and in vitro anti-inflammatory activity and antitumor potential of six ethanolic extracts of Bulgarian propolis samples, which were selected based on our previous research [16], to extend the information about their chemical composition and potential therapeutic effects.

## 2. Materials and Methods

### 2.1. Propolis

Fresh propolis samples, collected by beekeepers from six villages located in three districts of Bulgaria during 2022, were delivered to the laboratory by a courier (Table 1). The samples were labeled and stored in plastic containers at room temperature in darkness until analysis.

In the cytotoxicity test, a commercial ethanolic propolis extract under the trade name “Kleeva tinktura” (Higytest^®^, Sofia, Bulgaria) was used as a positive control.

### 2.2. Preparation of Propolis Extracts

The processing of raw propolis samples and the preparation of 70% ethanolic extracts (20 mg/mL) were carried out according to the method described in our previous work [16]. To conduct the anti-inflammatory and antitumor activity tests, the ethanol was vacuum-evaporated and the propolis extracts were redissolved in dimethylsulphoxide—DMSO (Carlo Erba Reagents SAS, Val de Reuil, France)—to the initial concentration.

### 2.3. Cell Culture

The highly invasive human breast cancer cell line MDA-MB-231 was a kind gift from Prof. Martin Berger from DKFZ, Heidelberg, Germany. The cell line was cultured in Dulbecco’s modified Eagle’s medium—DMEM (Sigma-Aldrich, St. Louis, MO, USA)—supplemented with 10% fetal bovine serum (FBS), L-glutamine (2 mM) and Penicillin (100 U/mL)/Streptomycin (100 µg/mL)/Amphotericin B (0.25 µg/mL). The cells were maintained in an incubator at 37 °C and 5% CO_2_ and were confirmed negative for mycoplasma by regular testing with a MycoStripTM mycoplasma detection kit InvivoGen (Toulouse, France).

### 2.4. Total Phenolic Content

The total phenolic content (TPC) of propolis extracts was assessed according to the method of Ivanov et al. [17] using the Folin–Ciocalteu reagent (Sigma-Aldrich, St. Louis, MO, USA). The results were expressed as mg equivalents of gallic acid (GAE)/g of extract.

### 2.5. Total Flavonoid Content

The total flavonoid content (TFC) of propolis extracts was evaluated following the method described by Ivanov et al. [17]. The results were expressed as mg of quercetin equivalents (QE)/g of extract.

### 2.6. Total Caffeic Acid Derivatives

The total caffeic acid derivatives (TCADs) of propolis extracts were determined by the method of Ivanov et al. [18] using Arnow’s reagent. The total caffeic acid derivatives were expressed as mg of caffeic acid equivalents (CAE)/g of extract.

### 2.7. High-Performance Liquid Chromatography (HPLC) Analysis of Phenolic Compounds

An HPLC unit Elite LaChrome (VWR™ Hitachi, Tokyo, Japan) equipped with a diode array detector (DAD) was used to determine the phenolic compounds in propolis extracts. The separation of the phenolic compounds was performed by Supelco Discovery^®^ HS C18 column (5 μm, 250 mm × 4.6 mm), operating at 30 °C under gradient conditions with a mobile phase consisting of 2% acetic acid (solvent A) and acetonitrile (solvent B). Caffeic, ferulic and 3,4-dihydroxybenzoic acids were used to build standard calibration curves with a linearity range of 10–100 µg/mL. The detection of phenolic compounds was carried out at 280 and 320 nm and with a flow rate of 0.8 mL/min. The results were expressed as mg/g of extract [19].

### 2.8. Antioxidant Activity

#### 2.8.1. DPPH Radical Scavenging Assay

The DPPH assay was performed by the method of Ivanov et al. [17] using DPPH (2,2-diphenyl-1-picrylhydrazyl) reagent (Sigma-Aldrich, St. Louis, MO, USA). The antioxidant activity was expressed as mM Trolox equivalents (TE)/g of extract. The IC_50_ values (half-maximal inhibitory concentration) were expressed as μg/mL of extract.

#### 2.8.2. Ferric-Reducing Antioxidant Power (FRAP) Assay

The FRAP assay was performed according to the method of Ivanov et al. [17] using 2,4,6-Tris(2-pyridyl)-s-triazine (TPTZ) (Sigma-Aldrich, St. Louis, MO, USA). The antioxidant activity was expressed as mM Trolox equivalents (TE)/g of extract.

### 2.9. In Vitro Anti-Inflammatory Activity (Inhibition of Albumin Denaturation)

The anti-denaturation assay was carried out according to Milusheva et al. [20]. The reaction mixture was prepared with 0.5 mL of 5% aqueous solution of human albumin (Albunorm 20, Octapharma AG, Brussels, Belgium) and 0.2 mL of the tested DMSO propolis extract, followed by incubation at 37 °C for 15 min. Next, each tube was filled with 2.5 mL of phosphate-buffered saline—PBS (pH 6.3), heated to 80 °C for 30 min, and then chilled for 5 min. A mixture of 2.5 mL of PBS and 0.2 mL of DMSO served as a blank, while the control sample contained 0.5 mL of albumin and 2.5 mL of PBS. The samples’ turbidity was measured at 660 nm. The percentage of inhibition of protein denaturation (% IPD) was calculated according to the following Equation (1):

(1) $$\%IPD=\frac{\left(Absorbance\;control\;-\;Absorbance\;sample\right)}{Absorbance\;control}\times 100$$

Using the same technique as for the propolis samples, the anti-inflammatory effect of the commercially available steroid anti-inflammatory drug Prednisolone Cortico (Antibiotic-Razgrad AD, Razgrad, Bulgaria) and non-steroid anti-inflammatory drug Aspirin (Bayer Bulgaria Ltd., Sofia, Bulgaria) at the same concentrations was ascertained for comparison.

All measurements (TPC, TFC, TCAD, antioxidant activity and anti-inflammatory activity) were performed on a UV/VIS spectrophotometer Camspec M107 (Spectronic-Camspec Ltd., Leeds, UK).

### 2.10. MTT Assay for Cell Cytotoxicity

The cytotoxic activity of propolis extracts towards breast cancer cells was analyzed by the 3-(4,5-Dimethyl-2-thiazolyl)-2,5-diphenyl-2H-tetrazolium bromide (MTT) assay measuring mitochondrial activity [21]. The cytotoxicity was tested at 24 h, 48 h and 72 h after treatment with the different propolis samples. MDA-MB-231 cells were seeded at a density of 5 × 10^4^ cells/mL for 24 h and 48 h and of 5 × 10^3^ cells/mL for 72 h in 96 well microplates. The cells were left to adhere for 24 h and then were treated with increasing concentrations of tested propolis extracts (from 10 to 150 µg/mL). The concentrations of extracts used in this study were in accordance with the literature data analyzing other types of propolis. Cells treated with DMSO at a concentration below 1% were used as a negative control.

The MTT assay was performed after treatment at the indicated time intervals. Briefly, 20 µL of MTT (M5655, Sigma-Aldrich, Taufkirchen, Germany) with a concentration of 5 mg/mL was directly added to the treated cells in 96 well microplates and incubated at 37 °C and 5% CO_2_ for 3 h. Next, the liquid was aspirated and 100 µL of 5% formic acid in isopropanol was added to dissolve the formed formazan crystals. The solution was well mixed by shaking, and the absorbance was measured immediately at 570 nm by a microplate reader (Tecan Infinite F200 PRO, Männedorf, Switzerland). The cell viability (%) was calculated as a percentage of the control using the following Equation (2):

(2) $$\%Cell\;viability=\frac{OD\;treated\;cells}{OD\;control\;cells}\times 100$$

To determine the values of the half-maximal inhibitory concentration (IC_50_), a non-linear regression analysis was utilized by GraphPad Prism version 5.0 (GraphPad Software Inc., La Jolla, CA, USA).

### 2.11. Statistical Analysis

Using statistical methods of MS Office Excel 2010 software, data from triplicate experiments were analyzed to determine the standard deviation (±SD) and the maximum estimation error at significant level *p* < 0.05.

## 3. Results and Discussion

### 3.1. Total Phenolic Content (TPC), Total Flavonoid Content (TFC), and Total Caffeic Acid Derivatives Content (TCADC)

In our previous work, we demonstrated that the high amounts of total polyphenolic and flavonoid compounds presented in propolis extracted with methanol contributed to its high antioxidant potential [16]. Although methanol is widely used as an extraction solvent due its higher polarity leading to high extract yield, its excessive toxicity restrains its further applications in cosmetics, food and medicine [22]. Hence, in this study, we selected the same propolis samples to determine whether the amounts of bioactive substances and antioxidant capacity changed after extraction of propolis with ethanol, which is considered as a low toxic solvent.

The amounts of total polyphenols in ethanolic propolis samples (TPC values from 190.04 to 317.0 mg GAE/g) (Table 2) were similar to those of the methanolic extracts of the same samples (TPC values from 188.50 to 737.27 mg GAE/g) observed in our previous work [16]. However, ethanolic extracts exhibited significantly lower TFC values (from 53.4 to 79.3 mg QE/g) compared to the methanolic ones (from 100.04 to 234.17 mg QE/g), probably due to the better extraction of the flavonoid compounds by methanol [16]. As seen from the results presented in Table 2, the ethanolic extract of propolis sample P4 and P2 exhibited the highest values of total phenolic content (TPC) and total flavonoid content (TFC), compared to the other propolis samples. In line with our results, Wang et al. [23] investigated 20 propolis samples extracted with ethanol from different regions of South Korea and reported values of TPC and TFC similar to ours. However, the propolis originating from Australia, Brazil and China examined in parallel with Korean samples by the same researchers showed lower TPC and TFC parameters as compared to our results. Segueni et al. [24] investigated Algerian and Turkish ethanolic propolis samples and stated that TPC values varied from 19.51 to 219.66 mg GAE/g, while TFC values varied between 5.27 and 74.57 mg QE/g, which were similar compared to our findings for Bulgarian propolis and other propolis originating from the Mediterranean region. Our results agreed with those reported by Aliyazıcıoglu et al. [25], who examined methanolic extracts of ten propolis samples from different locations of Turkey and determined that TPC values varied between 158.05 and 210.33 mg GAE/g.

The ethanolic propolis extracts were further evaluated for their total caffeic acid derivatives content (TCADC). Caffeic acid (3,4-dihydroxycinnamic acid) and its derivatives are phenolic compounds widely occurring in the plant tissues, poplar bud exudates and some biological products such as honey and propolis. As major bioactive compounds of propolis, they are responsible for its diverse pharmacological properties and therapeutic effects (antioxidant, anti-inflammatory, antiviral, anticoagulant, antitumor, neuroprotective and others) [26,27], which find wide medical application. The spectrophotometric quantification of TCADC in propolis extracts showed that samples P2 and P4 demonstrated the highest values of TCADC, which corresponded to the highest TPC and TFC values (Table 2). The presence of caffeic acid and its derivatives in the studied ethanolic propolis extracts was also confirmed by the HPLC assay (Table 3).

**Table 2:** Total phenolic content, total flavonoid content and total caffeic acid derivatives content of the ethanolic propolis extracts (20 mg/mL).

Propolis Sample	Total Phenolic Content, mg GAE/g of Extract	Total Flavonoid Content, mg QE/g of Extract	Total Caffeic Acid Derivatives, mg CAE/g of Extract
P1	226.8 ± 0.20	62.7 ± 0.10	8.2 ± 0.20
P2	301.3 ± 0.20	77.0 ± 0.10	12.1 ± 0.20
P3	190.4 ± 0.60	53.4 ± 0.03	5.9 ± 0.10
P4	317.0 ± 0.70	79.3 ± 0.06	11.6 ± 0.10
P5	233.0 ± 0.11 *	71.3 ± 0.10 *	9.7 ± 0.10
P6	243.4 ± 0.30	71.7 ± 0.10	8.9 ± 0.10

* According to our previous study [28].

**Table 3:** Phenolic compounds profile of the studied propolis samples.

Phenolic Compounds, mg/g of Extract	Propolis Samples					
	P1	P2	P3	P4	P5	P6
*Phenolic acids*						
Caffeic acid	3.16 ± 0.02	5.52 ± 0.00	3.46 ± 0.01	4.73 ± 0.00	4.91 ± 0.03	4.24 ± 0.04
p-coumaric acid	3.05 ± 0.03	3.97 ± 0.00	2.87 ± 0.01	4.96 ± 0.06	3.78 ± 0.00	3.30 ± 0.02
Sinapic acid	1.57 ± 0.00	2.27 ± 0.00	2.03 ± 0.02	4.05 ± 0.03	1.57 ± 0.01	1.69 ± 0.00
Caffeic acid benzyl ester	3.23 ± 0.03	4.05 ± 0.05	2.33 ± 0.06	5.13 ± 0.05	3.45 ± 0.02	4.18 ± 0.03
Cinnamic acid	0.86 ± 0.01	1.98 ± 0.04	0.28 ± 0.00	1.97 ± 0. 03	1.58 ± 0.01	1.15 ± 0.00
*Flavonoids*						
Isorhamnetin	0.91 ± 0.03	2.05 ± 0.02	1.32 ± 0.06	1.85 ± 0.05	1.83 ± 0.06	1.78 ± 0.01
Pinocembrin	9.41 ± 0.09	16.94 ± 0.10	8.17 ± 0.13	13.60 ± 0.11	20.24 ± 0.30	17.34 ± 0.35
Chrysin	29.71 ± 0.42	38.56 ± 0.46	42.59 ± 0.33	80.02 ± 0.55	64.61 ± 0.65	62.87 ± 0.32
Pinobanksin-3-O-propionate	30.56 ± 0.22	40.61 ± 0.18	25.39 ± 0.09	65.92 ± 0.51	46.87 ± 0.31	44.22 ± 0.12

### 3.2. Phenolic Compounds Profile

Propolis is a biological product that possesses many pharmacological activities due to the large number of different phenolic compounds. To gain more insight into the chemical composition of selected propolis extracts, we performed HPLC analysis. The results shown in Table 3 demonstrated the presence of five phenolic acids and four flavonoids in various ratios. Propolis samples P2 and P5 were characterized by the highest concentrations of caffeic acid, while samples P2 and P4 showed the highest content of sinapic acid, p-coumaric acid and cinnamic acid. Samples P4 and P6 exhibited the highest content of caffeic acid benzyl ester. Propolis samples P2 and P4 were characterized by the highest content of the flavonoid isorhamnetin, whereas samples P5 and P6 exhibited the highest concentration of the flavonoid pinocembrin. Samples P4 and P5 showed the highest concentration of the flavonoids chrysin and pinobanksin-3-O-propionate.

In ethanolic propolis samples originating from nine regions of Poland [29], the authors determined that the TPC ranged from 150.05 to 197.14 mg GAE/g, while the TFC varied between 35.64 and 62.04 mg QE/g, whose values were lower in comparison with our results (Table 2). And, vice versa, while in the propolis samples from Poland, the dominant phenolic acid was p-coumaric acid (37.54 to 116.95 mg/g), in our samples this component was found in very low concentrations (2.87 to 4.96 mg/g). Polish propolis samples also contained caffeic, ferulic, gallic, hydroxybenzoic and gentisic acids. Among the flavonoid compounds, chrysin and galangine were prevalent, while for two of the samples naringine was dominant. The values of chrysin were between 13.44 and 45.54 mg/g, which were close to our results. Consistent with our results were also those reported by Özkök et al. [30] for TPC values (34.53–259.40 mg GAE/g) of ethanolic propolis samples collected from 23 locations in Turkey. In the same study, authors identified four flavonoid compounds by HPLC (quercetin, galangin, apigenin and pinocembrin) and six phenolic acids (caffeic, p-coumaric, trans-ferulic, protocatechuic, trans-cinnamic and caffeic acid phenethyl ester—CAPE) in different ratios depending on the region of the sample collection. Using HPLC analysis, Aliyazıcıoglu et al. [25] examined methanolic extracts of ten propolis samples from different locations of Turkey and detected high concentrations of quercetin, benzoic acid, caffeic acid, ferulic acid and p-coumaric acid in all tested samples. Vanillic acid and chlorogenic acid were presented in minimal amounts in all samples; syringic acid, o-coumaric acid, epicatechin and rutin were found only in some samples, while the flavonoid catechin was not found in none of them.

Taken together, our results indicated that concentrations of bioactive compounds in propolis vary widely depending on the type of extraction solvent, extraction conditions as well as the specific climatic conditions of the geographic area, as previously confirmed by the literature data [31,32].

### 3.3. Antioxidant Activity

As previously noted, the antioxidant activity is a biological property that largely depends on the polyphenolic content. As known, polyphenols are natural exogenous antioxidant agents, which, when consumed with food, are absorbed unchanged or are degraded by the gut microbiota to various phenolic acids depending on the initial compound structure. The metabolites produced possess an unchanged structure of the units responsible for their antioxidant activity, exhibiting similar antioxidant properties and mechanisms of action to unaltered compounds [33].

The results in Table 4 show that the ethanolic propolis extracts exhibited variable but high values of antioxidant capacity determined by two independent methods—DPPH and FRAP. The highest values of the antioxidant activity were estimated for ethanolic propolis extracts P2, P4 and P6, which correlated with the higher amounts of TPC, TFC and TCADC, respectively, followed by P5, P1 and P3. It is worth mentioning that values for the antioxidant activity of all ethanolic propolis extracts remained relatively high (DPPH values from 1000.3 to 1606.0 mM TE/g; IC_50_ values from 29.80 to 47.14 μg/mL; FRAP values from 634.1 to 1134.5 mM TE/g) and similar to those of methanolic ones (DPPH values from 902.11 to 1464.75 mM TE/g and FRAP values from 758.80 to 1012.26 mM TE/g), as we have previously reported [16].

In contrast to our findings (Table 4), ethanolic propolis samples obtained from different regions of Poland exhibited very low antioxidant activity (DPPH values from 1.92 to 2.69 mM TE/g; FRAP values from 6.23 to 9.19 mM Fe(II)/g) [29]. Segueni et al. [24] investigated Algerian and Turkish ethanolic propolis extracts and stated that all propolis samples showed high levels of reducing power (determined by the FRAP assay) ranging from 267.3 to 2387.3 μmol TE/g compared to our data (Table 4). Aliyazıcıoglu et al. [25] examined methanolic extracts of ten propolis samples from different locations of Turkey and found that the values of antioxidant activity obtained by the FRAP method varied from 182.12 to 325.47 µM TE/g.

### 3.4. In Vitro Anti-Inflammatory Activity

The inflammation process is associated with denaturation of proteins. During this pathological process, secondary and tertiary structures of proteins are destroyed, due to which, their biological functions are then disturbed. The anti-inflammatory impact is closely linked to the capacity of certain substances—natural or synthetic—to lower the degree of inflammation [34]. It has been established that multiple drugs with anti-inflammatory action inhibit the thermally induced protein denaturation in a dose-dependent manner [35].

**Figure 1:**
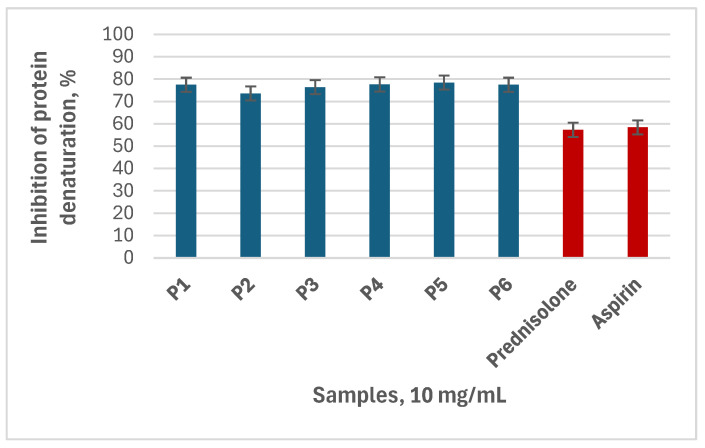
In vitro anti-inflammatory activity of the propolis extracts and the controls (10 mg/mL) expressed as inhibition of albumin denaturation (%).

The in vitro anti-inflammatory effect of the studied propolis extracts was determined as the inhibition of the thermally induced albumin denaturation. The results are presented as the percentage of inhibition of albumin denaturation (Figure 1).

The propolis sample P5 exhibited the highest anti-inflammatory effect, expressed as the highest inhibition of albumin denaturation (78.44 ± 2.21%), and the lowest IC_50_ value, followed by the samples P4 (77.66 ± 0.66%), P1 (77.5 ± 0.88%), P6 (77.5 ± 2.65%), P3 (76.41 ± 0.22%) and P2 (73.59 ± 1.55%). As seen from the obtained results, all ethanolic propolis extracts at a concentration of 10 mg/mL demonstrated similar values of albumin protection, which were higher than those of the conventional anti-inflammatory drugs used as controls (58.44 ± 0.44% for Aspirin and 57.34 ± 0.22% for Prednisolone Cortico) at the same concentration.

One of the most widely used and informative measures of a certain drug’s efficacy is the half-maximal inhibitory concentration (IC_50_). The anti-inflammatory activity of the studied propolis extracts expressed as IC_50_ values is presented in Table 5.

The non-steroidal and steroidal anti-inflammatory drugs (NSAIDs and SAIDs) are also effective in inhibiting inflammation, which, from a medical point of view, is a normal healing process; however, these drugs cause many adverse effects, especially with high dosages and prolonged intake. NSAIDs can cause gastric ulcers, stomach irritation and lower gastrointestinal disorders [36,37], while SAIDs increase the risk of hyperglycemia, predisposition to infections, peptic ulcer disease, glaucoma, cataracts, psychosis, depression, adrenal insufficiency, diabetes and osteoporosis [38]. Consequently, the results in the present study revealed a great anti-inflammatory effect of the propolis samples that can find application in the development of new medical formulations as alternatives to conventional anti-inflammatory drugs.

Despite the numerous publications on the in vivo anti-inflammatory effects of propolis, the scientific data on in vitro anti-inflammatory activity are very limited. Toutou et al. [39] used the method of inhibition of bovine serum albumin denaturation to assess the in vitro anti-inflammatory activity of seven propolis samples from different regions of Algeria. The authors determined that the values of the inhibition of the protein denaturation varied between 11% and 96%, as the maximum value was higher than the control ibuprofen applied at the same concentration. A study conducted by Araújo et al. [40] demonstrated that the hydroalcoholic Portuguese propolis extracts inhibited the denaturation of bovine serum albumin by 24.93% to 74.69%. Afonso et al. [41] evaluated the anti-inflammatory activity of Portuguese propolis ethanolic extracts and determined that they inhibited protein denaturation by 28% to 45%, and thus the values were lower than those obtained for the Bulgarian propolis samples in our research (minimal value of 73.59%). Consistent with our results were those obtained by Mendez-Encinas et al. [42], who determined that propolis extracts from Sonora state (Mexico) inhibited the heat-induced protein denaturation by 81.67–100% at concentrations from 6.25 to 50 µg/mL, while Diclofenac Sodium used as a reference drug inhibited the protein denaturation by 79.16–111.45% at the same concentration range.

### 3.5. In Vitro Antitumor Activity

Numerous studies have indicated that propolis obtained from different countries suppresses cell proliferation by inducing apoptosis and cell cycle arrest through multiple signaling pathways [43]. In this regard, we were interested in analyzing cell proliferation status after the treatment of highly invasive MDA-MB-231 breast cancer cells with the selected Bulgarian propolis samples at several time intervals (24 h, 48 h and 72 h). The in vitro cytotoxic activity of propolis extracts with varying concentrations (10–200 µg/mL) was tested by the MTT method (Table 6).

The obtained results showed that all propolis extracts reduced the proliferation of MDA-MB-231 breast cancer cells in a time- and a dose-dependent manner for 72 h (IC_50_ ranged between 9.24 and 21.22 µg/mL). Their high cytotoxicity was approved by using a positive control (“Kleeva tinktura”), which showed a significantly lower IC_50_ value (64.71 ± 6.95 µg/mL). In addition, the IC_50_ values of “Kleeva tinktura” were relatively close to those obtained for the positive control—a poplar type Bulgarian propolis extract (48.5 ± 5.7 µg/mL) used in our previous study [44], which confirmed the enhanced cytotoxic activity of the studied propolis samples.

As shown in Table 6, propolis samples exhibited the highest cytotoxic activity in the following order: P4, P6 and P5 at the 72-th h of incubation. It is worth noting that the ethanolic propolis extract P4 was characterized by the highest values of TPC, TFC and antioxidant capacity, which most probably contributed to the pronounced cytotoxic effect against metastatic breast cancer cells. In addition, based on the HPLC results, propolis extracts P4, P5 and P6 demonstrated the highest content of flavonoids chrysin and pinobanksin-3-O-propionate, whereas pinocembrin was detected in its highest concentrations in extracts P5 and P6 suggesting their role in the antitumor effect. In accordance with our assumption, pinocembrin, chrysin and pinobanksin-3-O-propionate have been found to exert strong anti-proliferative, growth-inhibitory and anti-invasive effects against various cancer cells and tumors [45,46,47]. Our results showed that the cytotoxic effect on the proliferation of human breast cancer cells correlated with the variability of the chemical composition of propolis samples from different regions of Bulgaria. The high levels of total polyphenols and, particularly, the dominant presence of caffeic acid benzyl ester as well as flavonoids chrysin, pinobanksin-3-O-propionate and pinocembrin in three of the propolis samples P4, P5 and P6 (from Gabrovo and Lovech regions) may explain their higher cytotoxic effect against MDA-MB-231 cells. In contrast, in our previous study [44], we found that the cytotoxicity of a propolis extract containing mainly terpenoids (di- and triterpenes) rather than polyphenols against MDA-MB-231 was almost twice as weak (IC_50_ ranged between 23 and 29 μg/mL for 72 h treatment), compared to the high-phenol propolis ethanol extracts used in the present study, emphasizing the crucial role of phenolic compounds in the anti-proliferative activity of propolis. Using the MTT assay, Mendez-Encinas et al. [42] confirmed the antitumor potential of Sonoran propolis extracts and their phenolic compounds chrysin, galangin and pinocembrin in the RAW 264.7 cell line. The authors stated that IC_50_ values of the extracts ranged between 26.5 and 49.4 μg/mL (depending on the propolis harvesting season), while the individual IC_50_ values for the three phenolic compounds were 56.2, 52.4 and 56.16 μg/mL, respectively. Abutaha et al. [48] determined by the MTT assay that propolis from China effectively reduced the viable counts of different cancer cell lines. The methanolic extract exhibited IC_50_ values as follows: for MDA-MB-231 (a breast cancer line)—74.12 μg/mL; LoVo (a colon cancer line)—74.12 μg/mL; HepG2 (a liver cancer line)—77.74 μg/mL; MCF7 (a breast cancer line)—95.10 μg/mL and A549 (a lung cancer line)—114.84 μg/mL), whereas the hexane extract showed IC_50_ values for the same cell lines of 52.11, 45.9, 52.11, 78.01 and 67.90 μg/mL, respectively.

The safety of utilizing ethanolic propolis extracts for normal cells has been proven by other authors. For instance, Mohamed et al. [49] have established the specificity and selectivity of propolis ethanolic extracts to cancer cells and their non-toxicity to normal cells. The estimated IC_50_ values for MCF10A (non-tumorigenic human mammary epithelial cell line) treated with ethanolic propolis extracts for 72 h were remarkably higher (72.10 ± 0.027 µg/mL) compared to IC_50_ values obtained for breast cancer cells. Moreover, it has been demonstrated that Bulgarian ethanolic propolis extract with high phenolic contents (similar to our propolis samples) was not cytotoxic towards human dermal fibroblasts (HDF) with IC_50_ values above 300 µg/mL (no dead cells were detected) [50].

These findings provide us with grounds to consider that the lower IC_50_ values of the studied Bulgarian ethanolic extracts that induce cytotoxicity in breast cancer cells would not be toxic to normal human cells.

## 4. Conclusions

The studied ethanolic extracts of propolis samples originating from three different regions in the northern part of Bulgaria exhibited high levels of the total phenols, total flavonoids and total caffeic acid derivatives, resulting in significant antioxidant activity. The propolis samples demonstrated promising in vitro anti-inflammatory activity, expressed as the inhibition of albumin denaturation, showing protection rates from 73.59% to 78.44%, which were significantly higher than those of the conventional non-steroidal and steroidal anti-inflammatory drugs Aspirin and Prednisolone Cortico used in the study. The propolis samples also showed pronounced in vitro antitumor potential against the highly invasive human breast cancer cell line MDA-MB-231, probably associated with the high amounts of the flavonoids chrysin, pinocembrin and pinobanksin-3-O-propionate. Based on the obtained results, we can conclude that investigated Bulgarian propolis is a high-quality beekeeping product, which can find successful practical application as an alternative anti-inflammatory remedy and a cosmetic ingredient. Furthermore, the studied propolis samples revealed significant biological and health beneficial properties with potential future application as food additives, nutritional value enhancers and natural preservatives, thus extending the shelf life of various food products.

## Figures and Tables

**Table 1:** Origin of the propolis samples.

Propolis Sample	Village	District/Region	GPS Coordinates
P1 (67) *	Gamzovo	Vidin	44°05′ N 22°45′ E
P2 (74)	Parsha	Gabrovo	42°57′ N 25°29′ E
P3 (75)	Ritya	Gabrovo	42°59′ N 25°25′ E
P4 (76)	Kozi rog	Gabrovo	42°57′ N 25°16′ E
P5 (77)	Burya	Gabrovo	43°02′ N 25°19′ E
P6 (79)	Malinovo	Lovech	42°90′ N 24°90′ E

* Numbers in the brackets indicate the numbering in our previous study [16].

**Table 4:** Antioxidant activity of the ethanolic propolis extracts (20 mg/mL).

Propolis Sample	Antioxidant Activity		
	DPPH, mM TE/g of Extract	IC_50_, μg/mL of Extract	FRAP, mM TE/g of Extract
P1	1127.7 ± 1.20	41.99 ± 0.46	708.4 ± 1.30
P2	1563.5 ± 0.90	30.57 ± 0.18	1062.8 ± 0.70
P3	1000.3 ± 0.30	47.14 ± 0.14	634.1 ± 1.00
P4	1606.0 ± 3.70	29.80 ± 1.03	1134.5 ± 1.20
P5	1407.1 ± 2.37 *	33.90 ± 1.26	950.6 ± 1.02 *
P6	1447.3 ± 2.20	32.97 ± 0.49	959.5 ± 1.20

* According to our previous study [28].

**Table 5:** Anti-inflammatory activity of the propolis extracts and the controls expressed as IC_50_.

Propolis Sample	IC_50_, mg/mL
P1	6.45 ± 0.07
P2	6.80 ± 0.14
P3	6.54 ± 0.02
P4	6.44 ± 0.05
P5	6.38 ± 0.18
P6	6.46 ± 0.22
Aspirin	8.56 ± 0.06
Prednisolone Cortico	8.72 ± 0.03

**Table 6:** IC_50_ values in MDA-MB-231 after exposure to different propolis extracts for 24 h, 48 h and 72 h. IC_50_ values are expressed as the mean ± SD from six repeats of two independent experiments.

Propolis Sample	IC_50_, µg/mL, 24 h	IC_50_, µg/mL, 48 h	IC_50_, µg/mL, 72 h
P1	107.3 ± 4.38	33.07 ± 3.41	19.65 ± 6.40
P2	92.44 ± 1.57	37.33 ± 10.13	15.31 ± 2.67
P3	140.3 ± 14.07	58.60 ± 7.96	21.22 ± 9.32
P4	60.56 ± 1.02	23.06 ± 4.17	9.26 ± 2.11
P5	73.78 ± 5.28	44.04 ± 5.04	13.62 ± 0.69
P6	60.71 ± 10.61	26.11 ± 3.20	9.24 ± 2.67
“Kleeva tinktura”	-	-	64.71 ± 6.95

## Data Availability

Datasets from the time of this study are available from the respective authors upon reasonable request.
